# Early Termination of Oncology Clinical Trials in the United States

**DOI:** 10.1002/cam4.5385

**Published:** 2022-10-28

**Authors:** Ellen Zhang, Steven G. DuBois

**Affiliations:** ^1^ Harvard Medical School Boston Massachusetts USA; ^2^ Dana‐Farber/Boston Children's Cancer and Blood Disorders Center Harvard Medical School Boston Massachusetts USA

## Abstract

**Purpose:**

The aim of this study was to evaluate the rate of early trial discontinuation of oncology trials and reasons for early termination, to assess potential trends in rates of oncology trial termination, and to perform a comprehensive analysis of predictors of early termination. This study intends to inform efforts in improving efficiency of the oncology clinical trial enterprise.

**Methods:**

We conducted a cross‐sectional study of interventional cancer clinical trials registered in ClinicalTrials.gov database from September 27, 2007 to June 30, 2015, with at least one site listed in the United States. We evaluated predictors of early trial termination using Fisher exact or *χ* ^2^ tests and logistic regression.

**Results:**

Of 8687 trials, 22.74% (*n* = 1975) were terminated trials. Rates of early trial termination appeared stable over the study. Statistically significant univariate predictors of early termination for any reason include cancer category, phase, funding source, location, and age. In multivariable analysis, trials spanning multiple cancer categories and international trials were less likely to terminate early whereas phase 2 trials and trials funded by academia/foundation were more likely to terminate early. The most common reason for early termination was “Other, Multiple Reasons, or Unknown” (36.9%), followed by accrual issues (34.5%). In multivariate analysis among all terminated trials, supportive care trials, phase 2 trials, and non‐industry funded trials had significantly higher odds of trial discontinuation specifically due to poor accrual.

**Conclusion:**

In this national sample of cancer clinical trials, early trial discontinuation was common. Many factors influenced early trial termination with poor accrual being a common reason. Specific trial features are associated with differential likelihood of early trial termination for any reason and for early trial termination due to poor accrual.

## Graphical abstract

Early discontinuation of oncology clinical trials is common, occurring in 22.7% of trials. The most common discrete reason for early termination of trials is due to accrual issues, with supportive care trials, phase 2 trials, and non‐industry sponsored trials at particular risk for early termination due to poor accrual.

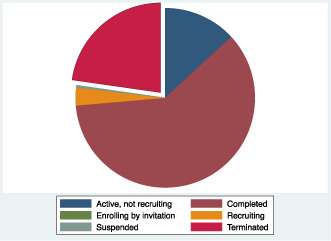

## 1 INTRODUCTION

Interventional clinical trials play a vital role in advancing new therapeutic approaches in medicine. Prior work has shown that a significant portion of clinical trials are terminated early.^1^, ^2^ Early termination can happen due to reasons that include, but are not limited to, poor accrual, funding issues, and emerging safety and/or efficacy signals. In the setting of safety or efficacy considerations, early termination may be appropriate, and this approach is commonly prespecified in the clinical trial statistical plan. In the case of early termination due to poor accrual, funding issues, or other logistical issues, early termination can result in utilization of resources without contributing knowledge to the scientific community. In these cases, there are ethical considerations for patients who enroll in clinical trials that do not reach a conclusion due to early termination.^3^

Despite the large and growing number of oncology clinical trials, there is limited research on early termination of cancer clinical trials. One analysis demonstrated that cancer trials have a higher likelihood of early termination when compared to trials in other disciplines.^4^ One preliminary report identified that poor accrual was the main reason for early trial termination in oncology, though the COVID‐19 pandemic was reported as a new additional reason.^5^ Another study showed that approximately one in six urologic oncology trials were terminated prematurely, with one in 10 closing early due to poor accrual.^6^

In this context, we conducted an analysis of publicly available data from ClinicalTrials.gov with the following aims. First, we sought to define the rate of early trial discontinuation of oncology trials as well as reasons for early termination. Second, we assessed potential trends in the rates of oncology trial termination. Third, we performed a comprehensive analysis of predictors of early termination, with a goal of informing efforts to improve the efficiency of the oncology clinical trials enterprise.

## 2 METHODS

### 2.1 Data source

We conducted a cross‐sectional study of interventional clinical trials for the treatment of patients with cancer and registered in ClinicalTrials.gov with at least one site listed in the United States. Trials that included conditions other than cancer, including cancer screening or prevention studies in people without cancer, were excluded. We limited our analysis by searching for cancer interventional studies registered from September 27, 2007 to June 30, 2015. This start date aligns with start of required registration of trials in ClinicalTrials.gov. This end date was chosen to allow sufficient time for trial completion/discontinuation status to be determined and reported in ClinicalTrials.gov. All trial statuses were included except those coded as “not yet recruiting” and “withdrawn” since these trials were registered but never accrued patients, as well as “unknown” given that the status was not verified within past 2 years. The ClinicalTrials.gov query was performed on a single day (September 1, 2021). This analysis of publicly available data did not involve human subjects and therefore institutional review board review was not required.

### 2.2 Variables

Definitions for data elements in ClinicalTrials.gov were used as per the Glossary of Common Site Terms and Clinical Trials.gov Protocol Data Element Definitions.^7^, ^8^ Trial entries in ClinicalTrials.gov provide details on the study population, condition, intervention type, start and completion dates, funding source, design characteristics, trial site(s), sex, age, phase, and current recruitment status.^9^ Investigators must periodically update these records.^9^ Certain data elements were further categorized for the purposes of analysis as described herein.

The condition under study was categorized as “solid tumor,” “hematological malignancy,” “CNS tumor,” or “multiple” if cancer type was not specified or if cancer spanned more than one of the previously mentioned cancer categories. As examples, if the eligible cancer types (e.g., breast and colon), all fell under only one of the aforementioned categories (e.g., solid tumor), then it was categorized as such; however, if the eligible cancer types included more than one of the categories (e.g., lung and glioma) or was histology‐agnostic (e.g., any cancer type with specific genomic feature regardless of primary site), then it was categorized as “multiple.” Interventions were categorized as “drug/biologic,” “radiation,” “procedure,” “behavior,” “device,” “bone marrow/stem cell transplant or other cell therapy,” “other,” and “multiple” if spanning multiple categories. The focus of the intervention category was the main experimental intervention being studied, oftentimes this would be compared to the current standard of care. Interventions were further categorized as “anticancer” if treating the cancer itself, or “supportive” if treating symptoms of cancer or side effects of cancer therapy. Study duration was calculated as the time between study start date and study completion date, the last visit where data was collected for any of the study outcomes. Trials were classified as “pediatric” if accepting only patients younger than 18, “adult” if only accepting those 18 and older, and “adult and pediatric” if it spanned 18 years old. If trials accepted only patients older than 60 years of age, it was also categorized as “geriatric.” Trial phases were categorized by the indicated phase, and if the trial spanned two phases (e.g., phase 1/2 trial), the trial was categorized as the lower phase.

Reasons for trial discontinuation were tabulated based on data provided in ClinicalTrials.gov and categorized as “accrual,” “funding,” “clinical efficacy,” “lack of clinical efficacy,” “side effects,” and “other” if the reason did not fit into any previous classification or no reason was cited.

### 2.3 Statistical considerations

Descriptive statistics for rates of trial termination were calculated. Fisher exact tests were used to evaluate the association between trial termination and categorical trial characteristics, except for trial characteristics with more than four categories in which case chi‐squared tests were used. Two sample *t*‐tests were used to compare continuous variables between terminated and non‐terminated trials. Two‐sided *p*‐values are reported with *p* < 0.05 and were considered statistically significant.

Logistic regression models were constructed to determine odds ratios for trial termination as a function of multiple trial characteristics, starting initially with all statistically significant variables from univariate testing and then removing variables that were no longer significant on multivariate testing to arrive at a final model containing only statistically significant variables.

All statistical analyses were performed in STATA BE version 17 (StataCorp).

## 3 RESULTS

### 3.1 Trial search outcome

**FIGURE 1:**
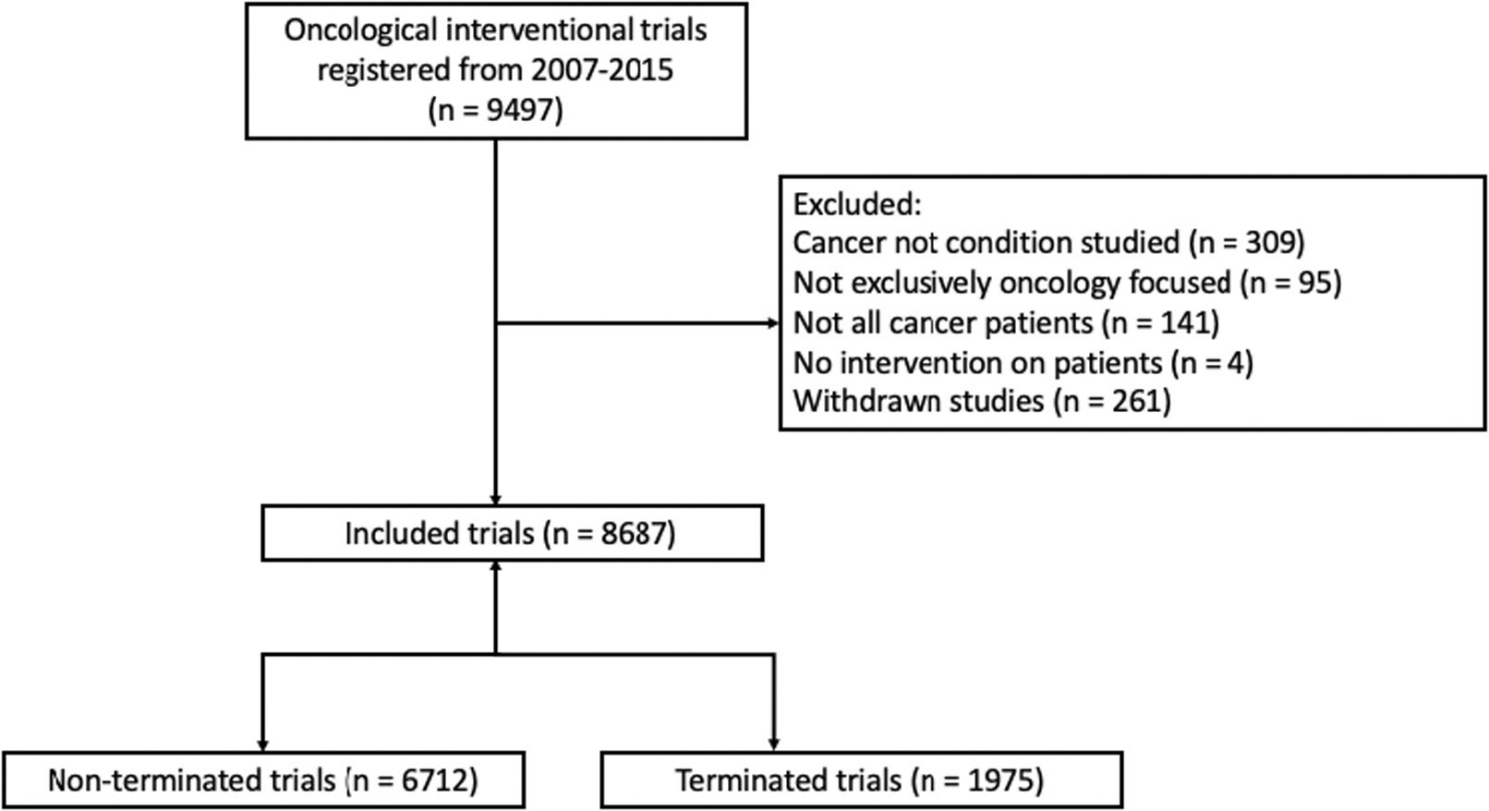
Flow diagram of trial inclusion and exclusion.

At the time of data collection, the ClinicalTrials.gov search yielded 9497 potential trials (Figure 1). Of these, 8.53% (*n* = 810) trials were excluded in which cancer was not the condition studied, trial was not exclusively cancer focused, not all patients had cancer, if there was no intervention, or if the status was “withdrawn.” The analytic cohort therefore included 8687 interventional cancer clinical trials.

### 3.2 Rates, reasons, and trends of early trial discontinuation

**FIGURE 2:**
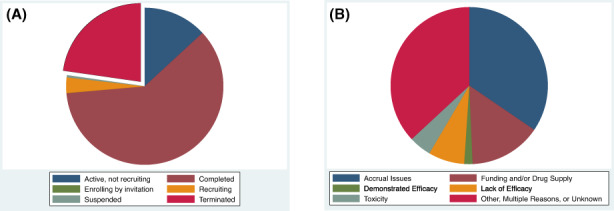
(A) Pie chart of trial statuses among 8687 interventional oncology trials. (B) Pie chart of reasons for early trial termination among 1975 trials terminated early.

Of 8687 trials, 77.3% (*n* = 6712) were non‐terminated trials and 22.7% (*n* = 1975) were terminated trials (Figure 2A). The most common reason for early termination was “Other, Multiple Reasons, or Unknown” in 36.9%, followed by accrual issues in 34.5% (Figure 2B). Only 1.7% of trials terminated early due to early evidence of efficacy, 7.4% terminated early due to early evidence of lack of efficacy, and 4.6% terminated early due to toxicity concern.

**FIGURE 3:**
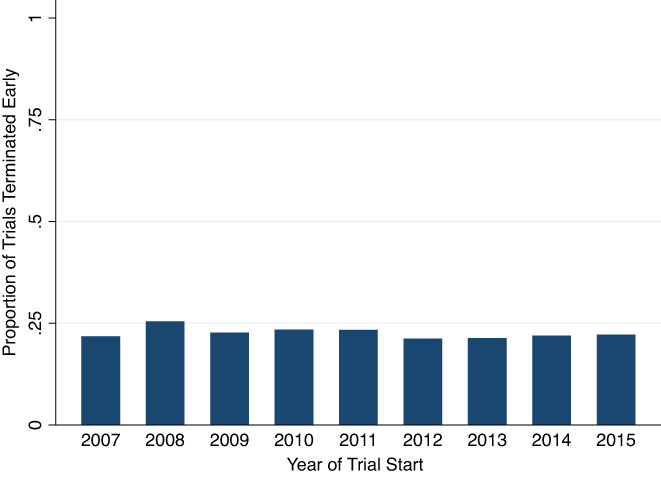
Trend in proportion of terminated trials over the study period (*n* = 8687 trials).

The rate of early trial termination over time appeared stable over the course of the study period from 2007 to 2015 (Figure 3). As expected, trials that were terminated early had lower mean enrollment (mean 124 vs. 48 patients; *p* < 0.001) and shorter duration (mean 3.3 vs. 5.7 years; *p* < 0.001).

### 3.3 Univariate predictors of trial discontinuation

Table 1 shows univariate predictors of trial discontinuation. Statistically significant predictors of early termination include cancer category (*p* = 0.011), phase (*p* < 0.001), funding source (*p* < 0.001), location (*p* < 0.001), and age (*p* = 0.016). Trials studying multiple cancer categories (e.g., patients with solid tumors and hematologic malignancies were eligible) had the lowest rate of early termination (17.1%) compared to trials that included patients just from one cancer category. Phase 3 trials had the lowest rate of early termination (18.5%) compared to other phases. Industry funded trials had the lowest rate of early termination (20.1%) compared to other funding categories. Trials located only in the United States were more likely to be terminated early (24.4%) compared to trials located both in the United States and internationally (16.8%). Trials that included patients <18 years of age had lower rates of early termination compared to trials that included only adults.

**TABLE 1:** Univariate predictors of early trial termination or early trial termination due to poor accrual

Trial characteristic	Proportion of trials terminated early (*n* = 8687)	*p*‐Value	Proportion of trials terminated early due to accrual issues among all terminated trials (*n* = 1975)	*p*‐Value
Cancer category				
Hematologic cancer	23.8%	0.011	35.5%	0.78
Solid tumor	22.9%		33.8%	
CNS tumor	21.5%		36.4%	
Multiple	17.1%		37.7%	
Therapeutic intent				
Anticancer	22.8%	0.86	33.6%	<0.001
Supportive care	22.3%		52.4%	
Intervention category				
Drug/biologic	22.4%	0.24	32.3%	<0.001
Radiation	25.2%		53.7%	
Procedure	22.7%		38.1%	
Behavioral	13.6%		25.0%	
Device	28.2%		45.5%	
Multiple	24.2%		43.8%	
Bone marrow/stem cell transplant or other cell therapy	27.2%		38.4%	
Other	24.8%		64.0%	
Design				
Randomized	21.2%	0.563	35.6%	0.012
Non‐randomized	20.4%		27.5%	
Sex				
Male & female	23.0%	0.32	33.5%	0.041
Male	20.9%		43.0%	
Female	21.3%		39.8%	
Age category				
Adult	23.1%	0.016	33.8%	0.024
Pediatric	13.5%		60.0%	
Adult & pediatric	19.2%		43.2%	
Geriatrics				
Geriatric	22.7%	0.558	34.6%	0.836
Not geriatric	25.0%		30.8%	
Phase				
1	21.2%	<0.001	28.6%	<0.001
2	25.4%		41.0%	
3	18.5%		26.8%	
4	25.6%		47.8%	
Funding source				
Industry	20.1%	<0.001	15.7%	<0.001
Government	20.9%		32.6%	
Other	27.1%		46.7%	
Multiple	23.2%		42.8%	
Start date				
2007–2009	23.8%	0.209	36.3	0.056
2010–2012	22.7%		36.2	
2013–2015	21.8%		30.7	
Start year				
2007	21.8%	0.385	40.9	0.130
2008	25.5%		37.9	
2009	22.7%		33.6	
2010	23.4%		39.3	
2011	23.4%		34.7	
2012	21.2%		34.1	
2013	21.4%		35.1	
2014	22.0%		27.6	
2015	22.2%		29.0	
Location				
USA	24.4%	<0.001	37.6%	<0.001
USA & International	16.8%		18.6%	

Since early termination due to poor accrual was the single most common discrete reason for early termination, we also evaluated predictors of early termination due to poor accrual among all terminated cancer clinical trials (Table 1). On univariate analysis, we found statistically significant predictors of trial discontinuation specifically due to poor accrual to include therapeutic intent (anticancer vs. supportive care; *p* < 0.001), intervention type (*p* < 0.001), randomization status (*p* = 0.012), age (*p* = 0.024), sex (*p* = 0.041), phase (*p* < 0.001), funding (*p* < 0.001), and location (*p* < 0.001). Of all trials terminated early, 33.6% of anticancer trials were terminated early due to accrual issues compared to 52.4% of supportive care trials. Radiotherapy trials had the highest rate of early termination due to accrual (53.7%) compared to other intervention types. Randomized trials had higher rates of termination due to accrual issues (35.6%) compared to non‐randomized trials (27.5%). Trials that included adults had lower rates of early termination due to accrual (33.8%) compared to trials that allowed patients <18 years. Meanwhile, trials that only included male participants had the highest rate of early termination due to accrual (43.0%) compared to terminated trials that only included females (39.8%) or those that included both males and females (33.5%). Reviewing phases, phase 4 (47.8%) and phase 2 (41.0%) trials had the highest rates of early termination due to accrual issues. Among terminated trials, industry funded trials had the lowest rate of termination due to accrual (15.7%) compared to other funding categories. Considering geography, trials located only in the US had higher rates of termination due to accrual (37.6%) compared to those located in both the US and internationally (18.6%).

### 3.4 Multivariable predictors of trial discontinuation

In multivariable analysis, cancer category, phase, funding, and location were significant independent predictors of trial termination for any reason (Table 2). The likelihood of early clinical trial termination was significantly lower for those investigating multiple cancer types than those investigating hematological cancers (odds ratio [OR] 0.70, 95% confidence interval [Cl] 0.54–0.91, *p* = 0.007). Phase 2 clinical trials were more likely to terminate early when compared to phase 1 (OR 1.27, 95% CI 1.14–1.41, *p* < 0.001). Trials funded by other sources were more likely to be terminated early than those funded by industry (OR 1.19, 95% CI 1.02–1.38, *p* = 0.025). Trials were less likely to be terminated if they were open internationally rather than open in the US alone (OR 0.65, 95% CI 0.55–0.76, *p* < 0.01).

**TABLE 2:** Multivariable predictors of early trial termination or early trial termination due to poor accrual

Trial characteristic	Odds Ratio (95% confidence interval)	*p*‐Value
Predictors of early trial termination for any reason (*n* = 8687)		
Cancer category		
Hematologic cancer	Reference	Reference
Solid tumor	0.96 (0.85–1.08)	0.46
CNS tumor	0.84 (0.66–1.08)	0.17
Multiple histology	0.70 (0.54–0.91)	0.007
Phase		
Phase 1	Reference	Reference
Phase 2	1.27 (1.14–1.41)	<0.001
Phase 3	1.05 (0.85–1.30)	0.62
Phase 4	1.24 (0.77–2.01)	0.38
Funding source		
Industry	Reference	Reference
Government	0.89 (0.72–1.10)	0.27
Other	1.19 (1.02–1.38)	0.03
Multiple	0.96 (0.84–1.10)	0.55
Trial location		
US only	Reference	Reference
US plus international site	0.65 (0.55–0.76)	<0.001
Predictors of early trial termination for poor accrual (*n* = 1975)		
Phase		
Phase 1	Reference	Reference
Phase 2	1.58 (1.29–1.94)	<0.001
Phase 3	1.39 (0.90–2.15)	0.13
Phase 4	2.41 (0.99–5.91)	0.05
Funding source		
Industry	Reference	Reference
Government	2.68 (1.77–4.07)	<0.001
Other	4.56 (3.41–6.08)	<0.001
Multiple	3.87 (2.96–5.08)	<0.001
Therapeutic intent		
Anticancer	Reference	Reference
Supportive care	1.71 (1.13–2.60)	0.011

Among all terminated cancer clinical trials, we also performed multivariate analysis to identify predictors of trial discontinuation specifically due to poor accrual (Table 2). Supportive care trials were more likely to be terminated due to poor accrual than anticancer trials (OR 1.71, 95% CI 1.13–2.60, *p* = 0.011). Phase 2 trials were more likely to be terminated due to accrual issues compared to phase 1 (OR 1.58, 95% CI 1.29–1.94, *p* < 0.001). Trials funded by sources other than industry were more likely to be terminated due to accrual issues than those funded by industry (ORs >2.6 and *p* < 0.001 for all other funding sources).

## 4 DISCUSSION

Our study demonstrates that among cancer interventional trials, early trial discontinuation is common. Over the course of study period from 2007 to 2015, the rate of early trial termination remained stable, with overall 22.7% of oncology trials terminating early. Statistically significant predictors of early termination include cancer category, phase, funding source, location, and age. In multivariable analysis, cancer category, phase, funding, and location were significant independent predictors of trial termination for any reason. Early termination due to toxicity, early efficacy, or early lack of efficacy accounted for the minority of trials that were terminated early. In contrast, poor accrual was a common reason for early termination, accounting for 34.5% of terminated trials. Statistically significant predictors of trial discontinuation specifically due to poor accrual includes therapeutic intent, intervention type, age, gender, phase, funding, and location. In multivariate analysis, therapeutic intent, phase, and funding were significant independent predictors of trial discontinuation specifically due to poor accrual.

Our early termination rate of 22.7% for oncology trials is higher than those in other disciplines. Reviewing cardiovascular clinical trials, 10.9% were terminated prematurely.^10^ Meanwhile, pregnancy related trials found that 6.1% of trials were terminated.^11^ Orthopedic trials had an early termination rate of 7.7% for shoulder‐related, 12.7% elbow‐related, and 14.0% spine‐related. The only other discipline with termination rates comparable to those of oncology trials is pediatrics where 19% of trials have been reported to discontinue early.^12^

Key independent trial characteristics associated with higher rates of early termination in oncology included phase 2 trials, non‐industry funded, and US‐based only. In contrast, histology agnostic trials that spanned disease groups had lower rates of early termination. These findings are consistent with those from other disciplines. One study on cardiovascular clinical trials found that trial termination predictors included location, intervention type, phase, therapeutic intent, and year of initiation.^13^ In obstetrics, independent predictors of trial termination included number of study locations, available results, study type, randomized design, study purpose, intervention type (drug or nondrug), and study location (including locations outside USA).^11^ In orthopedics, industry sponsored trials, phase 2 trials, blinded trials, and device trials appear to be associated with higher rates of early termination.^14^, ^15^ In contrast, an evaluation of pediatric trials showed that industry funding was associated with lower rates of early termination.^12^ While differing variables serve as predictors for clinical trials termination across disciplines, the factors identified herein should be considered as risk factors for early termination during early development of oncology trials.

The most common single reason for cancer clinical trial early termination was poor accrual, which is also a common finding found in other disciplines. A 2015 cross‐sectional study of terminated clinical trials on ClinicalTrials.gov found that 39% of all terminated trials were due to accrual.^1^ A 2014 study found that cardiovascular clinical trials were more likely to be terminated due to poor accrual (53.6%),^10^ which was also supported by a 2017 study (41%).^13^ The 2014 study on cardiovascular clinical trials found that mixed‐source founding and university/hospital funding were independently associated with a higher risk of study termination due to low recruitment; meanwhile, NIH/US federal funding, behavior/diet intervention, and single‐arm design were factors independently associated with lower risk for early termination due to low recruitment.^10^ Studies of obstetrics and orthopedic trials likewise show that accrual difficulty is one of the most frequently cited reason for early termination.^11^, ^14^ Taken together with the available literature, our findings suggest that study teams need to develop more realistic accrual goals and robust recruitment strategies. In oncology, this appears to be particularly critical for supportive care trials, trials funded by sources other than industry, and phase 2 and phase 4 trials. Of note, phase 4 trials are often post‐marketing studies and therefore patients may be able to access the therapy under investigation through commercial mechanisms. These trials had the highest odds ratio for early termination due to poor accrual, which further emphasizes that recruiting patients can be difficult given standards of care that can already be in place or rapidly evolving.

Several limitations should be noted when interpreting our findings. Given that this study analyzed only trials in ClinicalTrials.gov, it must be considered that there might be additional cancer clinical trials not captured in our analysis, especially phase I clinical trials.^16^ The rate of nonregistration of cancer clinical trials is not known, but it is unlikely that these trials have higher rates of completion given federal and editorial policies mandating registration. Another limitation is that our cancer categories are quite broad, in the context of this pan‐cancer analysis. Our goal in categorizing cancer types into these board categories was to investigate possible patterns that might be associated with higher rates of early termination. In addition, our analysis depends on the accuracy of trial data provided to ClinicalTrials.gov by investigators and sponsors. This issue is mitigated in part by automated data validity checks and manual review of ClinicalTrials.gov to ensure data accuracy before public posting.^17^ Concurrently, there were missing data in the registry such as trial phases and reasons for discontinuation, which is mitigated by the robust size of the data set.

Ultimately, we have found that cancer clinical trials are frequently discontinued early. As most cases of early termination are due to accrual and other operational issues, our study highlights the considerable inefficiency and waste associated with early termination of oncology trials. Our work highlights trial characteristics that merit a focused effort to support adequate accrual and study teams need to plan for drug supply, adequate funding, and other operational issues to avoid early termination for reasons other than for safety and/or efficacy reasons. Prior planning to reduce likelihood of early termination is of paramount importance as there are important ethical concerns associated with patients participating in trials that will not contribute to greater scientific knowledge. Monitoring this issue and routinely recording reason for trial termination will allow our findings to be revisited in future studies. Future opportunities for research include reviewing patterns of early termination specifically in trials for patients with common histologies, examining long‐term impacts of the COVID‐19 pandemic on oncology trial early termination, and exploring termination rates internationally. Ultimately, although there have been policies and interventions implemented to both increase the number of cancer clinical trials and improve trial reporting, there needs to be further consideration and action to ensure that patient participation in cancer trials advances the field.

## AUTHOR CONTRIBUTIONS

**Ellen Zhang:** Conceptualization (equal); data curation (equal); formal analysis (equal); investigation (equal); methodology (equal); supervision (equal); validation (equal); writing – original draft (equal); writing – review and editing (equal). **Steven G. DuBois:** Conceptualization (equal); data curation (equal); formal analysis (equal); funding acquisition (lead); investigation (equal); methodology (equal); project administration (lead); resources (equal); software (equal); supervision (lead); validation (equal); visualization (equal); writing – original draft (equal); writing – review and editing (equal).

## FUNDING INFORMATION

Supported by Alex's Lemonade Stand Foundation Center of Excellence award (SGD).

## DISCLOSURES

SGD reports consulting fees from Amgen, Bayer, Jazz, and Loxo, and travel expenses from Loxo Oncology, Roche, and Salarius.

## Data Availability

The data that support the findings of this study are publicly available in ClinicalTrials.Gov at https://www.clinicaltrials.gov/.
